# Author Correction: The paradox of second-order homophily in networks

**DOI:** 10.1038/s41598-021-96670-4

**Published:** 2021-08-20

**Authors:** Anna Evtushenko, Jon Kleinberg

**Affiliations:** 1 Department of Information Science, Cornell University, Ithaca, NY USA; 2 Department of Computer Science, Cornell University, Ithaca, NY USA

Correction to: *Scientific Reports* 10.1038/s41598-021-92719-6, published online 25 June 2021

The original version of this Article contained errors.

In the section “Relationship to the Friendship Paradox”,

“Specifically, in the Facebook100 networks the correlation between degree and first-order homophily is generally between − 0.2 and 0.25.”

now reads:

“Specifically, in the Facebook100 networks the correlation between degree and first-order homophily is generally between −0.25 and 0.2.”

The original Article has been corrected.

