# Author Correction: The paradox of second-order homophily in networks

**DOI:** 10.1038/s41598-022-23028-9

**Published:** 2022-11-02

**Authors:** Anna Evtushenko, Jon Kleinberg

**Affiliations:** 1 Department of Information Science, Cornell University, Ithaca, NY USA; 2 Department of Computer Science, Cornell University, Ithaca, NY USA

Correction to: Scientific Reports 10.1038/s41598-021-92719-6, published online 25 June 2021.

The Supplementary Information published with this Article contained errors.

Firstly, in the second equation of the “1 Step-by-step proof of the list version” section, the original index ‘B’ was incorrectly given as ‘R’.

Secondly, the following argument for why the denominator has to be positive was added: “all homophily values are between 0 and 1”.

Thirdly, the sum of the two terms ‘T_ij_ + T_ji_’ was incorrectly given as ‘T_ij_’.

Finally, the argument for why “the red gap for the list version with homophily diversity in red nodes is positive” has been simplified.

The original Supplementary Information file is provided below.

These errors have now been corrected in the Supplementary Information file that accompanies the original Article.

## Supplementary Information

Supplementary Information.

