# Supplementary material for: Author Correction: The paradox of second-order homophily in networks
Source: Sci Rep. 2022 Nov 2;12:18507. doi: 10.1038/s41598-022-23028-9 (PMC9630323; doi:10.1038/s41598-022-23028-9)
Supplement: Supplementary file 1 — Supplementary Information. [file 41598_2022_23028_MOESM1_ESM.pdf]

The Paradox of Second-Order Homophily in Networks

Anna Evtushenko,[1,3*] Jon Kleinberg[1,2,3]

[1]Department of Information Science
[2]Department of Computer Science
[3]Cornell University, Ithaca NY, USA

*anna@infosci.cornell.edu

Supplementary Information

Contents

1 Step-by-step proof of the list version **2**

2 Step-by-step proof of the special case of the singular version **5**

3 Constructing a two-type network from parameters: double configuration model **9**

4 Simulation **10**

5 Full derivation of list version gap size formula for a special case **11**

6 Examples where the Friendship and the Homophily Paradoxes co-occur and don't **15**

1 Step-by-step proof of the list version

Let's say a red node i has homophily h_i and degree d_i. This means it has $h_i d_i$ red friends and $(1-h_i)d_i$ blue friends, and, when i is treated as a friend of those nodes for the list version, it will be counted $h_i d_i$ times in the red histogram and $(1-h_i)d_i$ times in the blue histogram.

Given: all $h_i \in [0,1]$. Not all red h_i are equal (there is homophily diversity in red nodes). There are n total nodes, and k of them are red.

Let's represent the means of the red and blue histograms by counting each red node's homophily as a separate term (some h_i's may be equal but that's okay).

$$\mu_R^{(R)} = \frac{\sum_{i=1}^{k} d_i h_i^2}{\sum_{i=1}^{k} d_i h_i}$$

$$\mu_R^{(R)} = \frac{\sum_{j=1}^{k} d_j (1-h_j) h_j}{\sum_{j=1}^{k} d_j (1-h_j)}$$

We'd like to prove that $\mu_R^{(R)} - \mu_B^{(R)} > 0$, or

$$\frac{\sum_{i=1}^{k} d_i h_i^2}{\sum_{i=1}^{k} d_i h_i} - \frac{\sum_{j=1}^{k} d_j (1-h_j) h_j}{\sum_{j=1}^{k} d_j (1-h_j)} > 0$$

Let's bring this to the common denominator and remove it because the denominator of each fraction is positive (as at least one homophily value is positive, at least one < 1, and all degrees are positive).

$$\sum_{i=1}^{k} d_i h_i^2 \sum_{j=1}^{k} d_j (1-h_j) - \sum_{i=1}^{k} d_i h_i \sum_{j=1}^{k} d_j (1-h_j) h_j > 0$$

Rearranging,

$$\sum_{i=1}^{k}\sum_{j=1}^{k} d_i h_i^2 d_j (1-h_j) - \sum_{i=1}^{k}\sum_{j=1}^{k} d_i h_i d_j (1-h_j) h_j > 0$$

We can now find common terms to get

$$\sum_{i=1}^{k}\sum_{j=1}^{k} d_i d_j h_i (1-h_j)(h_i - h_j) > 0$$

First note that if $h_i = h_j$ the term is 0.

Then note that if $h_i \neq h_j$, at least one of the terms for (i, j) and (j, i) is not zero.

Proving that by contradiction:

If the above is wrong, with algebra we get:

1. $h_i(1 - h_j) = 0$
2. $h_j(1 - h_i) = 0$
3. $h_i \neq h_j$

From (1), $h_i = 0$ or $h_j = 1$. From (2), $h_j = 0$ or $h_i = 1$. If $h_i = 0$ to satisfy (1), h_i can't be 1 for (2), so h_j has to be 0 to satisfy (2). Then $h_i = h_j$. Similarly if $h_j = 1$ to satisfy (1) h_j can't be 0 for (2) and h_i has to be 1 to satisfy (2). Then $h_i = h_j$. Contradiction.

We know from homophily diversity in red nodes that there is at least one pair (i, j) such that $h_i \neq h_j$. (If there is no homophily diversity in red nodes, the sum is 0.)

Next, we'd like to prove that for all such pairs (i, j), the sum of the two terms for (i, j) and (j, i), T_{ij}, is positive.

$$T_{ij} = d_i d_j h_i (1 - h_j)(h_i - h_j)$$

$$T_{ji} = d_j d_i h_j (1 - h_i)(h_j - h_i)$$

$$T_{ij} + T_{ji} > 0$$

$$d_i d_j h_i (1 - h_j)(h_i - h_j) + d_j d_i h_j (1 - h_i)(h_j - h_i) > 0$$

d_i and $d_j > 0$ so

$$h_i(1 - h_j)(h_i - h_j) + h_j(1 - h_i)(h_j - h_i) > 0$$

$$h_i(1 - h_j)(h_i - h_j) - h_j(1 - h_i)(h_i - h_j) > 0$$

$$(h_i(1 - h_j) - h_j(1 - h_i))(h_i - h_j) > 0$$

Suppose $h_i > h_j$ so $h_i - h_j > 0$.

$$h_i(1 - h_j) - h_j(1 - h_i) > 0$$

$$h_i - h_i h_j - h_j + h_j h_i > 0$$

$$h_i - h_j > 0$$

This is true.

Going back to before assumption:

$$(h_i(1-h_j) - h_j(1-h_i))(h_i - h_j) > 0$$

Now suppose $h_i < h_j$ so $h_i - h_j < 0$.

$$h_i(1-h_j) - h_j(1-h_i) < 0$$

$$h_i - h_i h_j - h_j + h_j h_i < 0$$

$$h_i - h_j < 0$$

This is true.

Since terms for $h_i = h_j$ are 0 and pair-terms for $h_i \neq h_j$ exist and are positive, the whole sum is positive, so the red gap for the list version with homophily diversity in red nodes is positive.

2 Step-by-step proof of the special case of the singular version

We start with the observation that the second-order red homophily of a red node i with homophily h_i and degree d_i is equal to the first-order homophily of a random red friend of theirs.

Let the nodes be partitioned into a set R of k red nodes and a set B of $n-k$ blue nodes. For clarity, let a red node's first-order homophily be denoted h_i, and let a blue node's first-order homophily be denoted p_i. Similarly, let a red node's degree be d_i and a blue node's degree be c_i. Let $N(i)$ denote the neighbors of a node i, and for two sets of nodes, let $E(S,T)$ be the set of edges with one end in S and the other end in T. If we first choose a random red node i

and then choose a random red friend from among their $h_i d_i$ red friends, the expected first-order homophily we see, which is equal to mean second-order red homophily of red nodes, is

$$\mu_R^{(R,\text{sing})} = \frac{1}{k}\sum_{i\in R}\left(\frac{1}{h_i d_i}\sum_{j\in R\cap N(i)} h_j\right)$$

Rewriting to see how each h_i is counted with i being a red friend of red nodes:

$$\mu_R^{(R,\text{sing})} = \frac{1}{k}\sum_{i\in R}\left(h_i\sum_{j\in R\cap N(i)} \frac{1}{h_j d_j}\right)$$

There is a third way to represent this sum, too. Adapting an argument that Kramer et al. used in the different context of the Friendship Paradox (*1*), the $\mu_R^{(R,\text{sing})}$ sum runs over each edge in $E(R,R)$ twice, once in the outer sum for i and once in the outer sum for j. Thus the expression is also equal to

$$\mu_R^{(R,\text{sing})} = \frac{1}{k}\sum_{\{i,j\}\in E(R,R)}\left(\frac{h_j}{h_i d_i}+\frac{h_i}{h_j d_j}\right)$$

We can apply a similar approach to a random red friend of a random blue node. If we first choose a random blue node i and then choose a random red friend from among their $(1-h_i)d_i$ red friends, the expected first-order homophily we see, which is equal to mean second-order red homophily of blue nodes, is

$$\mu_B^{(R,\text{sing})} = \frac{1}{n-k}\sum_{i\in B}\left(\frac{1}{(1-p_i)c_i}\sum_{j\in R\cap N(i)} h_j\right)$$

Again, we can rewrite this to see how each h_i is counted with i being red a friend of blue nodes:

$$\mu_B^{(R,\text{sing})} = \frac{1}{n-k}\sum_{i\in R}\left(h_i\sum_{j\in B\cap N(i)} \frac{1}{(1-p_j)c_j}\right)$$

For our special case, assume:

- $\forall i \in R, d_i = d$
- $\forall j \in B$, their degree $c_j = c$

- $\forall j \in B$, their homophily $p_j = p$
- no restriction on h_i besides $h_i \in [0, 1]$, no restriction on the relationship between d and c, no restriction on k as it relates to n, no restriction on n.

Let's first prove that $\mu_R^{(R,\text{sing})} > \frac{1}{k}\sum_{i \in R} h_i = \lambda_R$.

From above

$$\mu_R^{(R,\text{sing})} = \frac{1}{k} \sum_{\{i,j\} \in E(R,R)} \left(\frac{h_j}{h_i d} + \frac{h_i}{h_j d} \right)$$

And $\lambda_R = \frac{1}{k}\sum_{i \in R} h_i$

$$\frac{1}{k} \sum_{\{i,j\} \in E(R,R)} \left(\frac{h_j}{h_i d} + \frac{h_i}{h_j d} \right) = \frac{1}{kd} \sum_{\{i,j\} \in E(R,R)} \left(\frac{h_j}{h_i} + \frac{h_i}{h_j} \right) > \frac{1}{kd} \sum_{\{i,j\} \in E(R,R)} 2$$

Above, the inequality for reciprocals can be strict because there is homophily diversity in red nodes. Notice that the number of red edges $|E(R, R)|$ is

$$\frac{1}{2} \sum_{i \in R} h_i d$$

Then:

$$\frac{1}{kd} \sum_{\{i,j\} \in E(R,R)} 2 = \frac{1}{kd} \left(\frac{1}{2} \sum_{i \in R} h_i d \right) 2 = \frac{1}{k} \sum_{i \in R} h_i = \lambda_R$$

Thus $\mu_R^{(R,\text{sing})} > \lambda_R$ in this special case.

Now, let's see how p relates to λ_R. First, we know from the balance equation that,

$$\sum_{i \in R} d_i(1 - h_i) = \sum_{j \in B} c_j(1 - p_j)$$

Or, in our case,

$$\sum_{i \in R} d(1 - h_i) = \sum_{j \in B} c(1 - p_j)$$

We'll compute mean $p_i = \overline{p}$ right now and equate it to p at the end. From the above,

$$dk - d \sum_{i \in R} h_i = c(n - k) - c \sum_{j \in B} p_j$$

$$dk - dk\frac{1}{k}\sum_{i\in R} h_i = c(n-k) - c(n-k)\frac{1}{n-k}\sum_{j\in B} p_j$$
$$dk - dk\lambda_R = c(n-k) - c(n-k)\overline{p}$$

$$dk(1-\lambda_R) = c(n-k)(1-\overline{p})$$
$$\frac{dk}{c(n-k)}(1-\lambda_R) = 1-\overline{p}$$
$$\overline{p} = 1 - \frac{dk}{c(n-k)}(1-\lambda_R)$$
$$\overline{p} = \frac{cn - (c+d)k + dk\lambda_R}{c(n-k)}$$

Here, $\overline{p} = p$.

Going back to $\mu_B^{(R,\text{sing})}$, which is

$$\mu_B^{(R,\text{sing})} = \frac{1}{(n-k)c}\sum_{i\in B}\left(h_i \sum_{j\in R\cap N(i)} \frac{1}{(1-p)}\right)$$

We get,

$$\mu_B^{(R,\text{sing})} = \frac{d}{(n-k)c}\frac{1}{(1-p)}\sum_{i\in B} h_i(1-h_i)$$

From what we know about p,

$$\frac{1}{(1-p)} = \frac{1}{1-\dfrac{cn-(c+d)k+dk\lambda_R}{c(n-k)}} = \frac{1}{\dfrac{c(n-k)-cn+(c+d)k-dk\lambda_R}{c(n-k)}} = \frac{c(n-k)}{dk-dk\lambda_R} = \frac{c(n-k)}{dk(1-\lambda_R)}$$

Hence

$$\mu_B^{(R,\text{sing})} = \frac{d}{(n-k)c}\frac{c(n-k)}{dk(1-\lambda_R)}\sum_{i\in B} h_i(1-h_i) = \frac{1}{k(1-\lambda_R)}\sum_{i\in B} h_i(1-h_i)$$

We already know,

$$\mu_R^{(R,\text{sing})} > \frac{1}{k}\sum_{i\in R} h_i = \lambda_R$$

Now, to prove $\mu_R^{(R,\text{sing})} > \mu_B^{(R,\text{sing})}$, we need to show,

$$\lambda_R \geq \mu_B^{(R,\text{sing})} = \frac{1}{k(1-\lambda_R)}\sum_{i\in B} h_i(1-h_i)$$

Multiplying by $(1-\lambda_R)$ and expanding λ_R we get,

$$\lambda_R(1-\lambda_R) \geq \frac{1}{k}\sum_{i\in B} h_i(1-h_i)$$

$$\left(\frac{1}{k}\sum_{i\in R} h_i\right)\left(1-\frac{1}{k}\sum_{i\in R} h_i\right) \geq \frac{1}{k}\sum_{i\in B} h_i(1-h_i)$$

$$\left(\frac{1}{k}\sum_{i\in R} h_i\right)\left(\frac{1}{k}\sum_{i\in R}(1-h_i)\right) \geq \frac{1}{k}\sum_{i\in B} h_i(1-h_i)$$

Now, relabel h's so that $h_i \geq h_j$ if $i > j$. This means that $(1-h_i) \leq (1-h_j)$ for $i > j$. Now, the last statement is true by (reverse) Chebyshev's sum inequality.

3 Constructing a two-type network from parameters: double configuration model

To create a network of red and blue nodes with parameters n, k, d, c, homophily samples $h_1, ...h_k$ and $p_{k+1}, ...p_n$ set or calculated as described in the discussion of simulation and gap size prediction below, we use what we call a double configuration model. For each red node, we take their homophily h_i, multiply it by d, and round it to get its within-type degree d_{iw}. We subtract it from d to get its outside-type degree d_{io}. We collect d_{iw}'s into a degree sequence and create the red subgraph with this degree sequence using the configuration model algorithm. We perform analogous operations on blue nodes (using p_j and c) to calculate c_{jw}'s and c_{jo}'s and to create the blue subgraph.

We then sum d_{io}'s and c_{jo}'s ($\sum_{i\in R} d_{io}, \sum_{j\in B} c_{jo}$), both of which are the number of edges between types, and see if they equal each other, as they must. If they don't, we look at the sum that's larger, get a random node from that type, decrease its outside-type degree by 1, and repeat the comparison of the sums, until they are equal. In the simulated example just below, they were equal right away.

Once the sums are equal, we take each red node i and put d_{io} copies of it into a new set R_o. We take each blue node j and put c_{jo} copies of it into a new set B_o. We permute both sets, create a ranking for them based on the permutation and create edges between the elements of the two sets if their rankings are equal. In the end, the copies of red node i would get d_{io} pairings to blue nodes, just like node i should. We take the information on which red node copy is connected to which blue node copy in this scenario, and connect their original nodes between the red subgraph and the blue subgraph that we have, getting the final graph.

4 Simulation

Here, we'd like to show that the result holds in the most symmetrical contexts and in data. We have proven the singular version result for the case with no homophily diversity in blue nodes, so in the simulation below we'll use the following parameters: $n = 10000, k = 5000, d = 100$ (same for all red nodes); half of the red nodes would have homophily 0.6, the other would have homophily 0.4, and the same would be true for blue nodes. We'll calculate the common degree of blue nodes c from the balance equation (we round c to make it integer). Because of the symmetry of the situation, it would also be equal to 100. Then, we create the network via double configuration model as described above and compute the red and the blue gaps for both the list and the singular versions of second-order homophily. The results are presented in Figure S1.

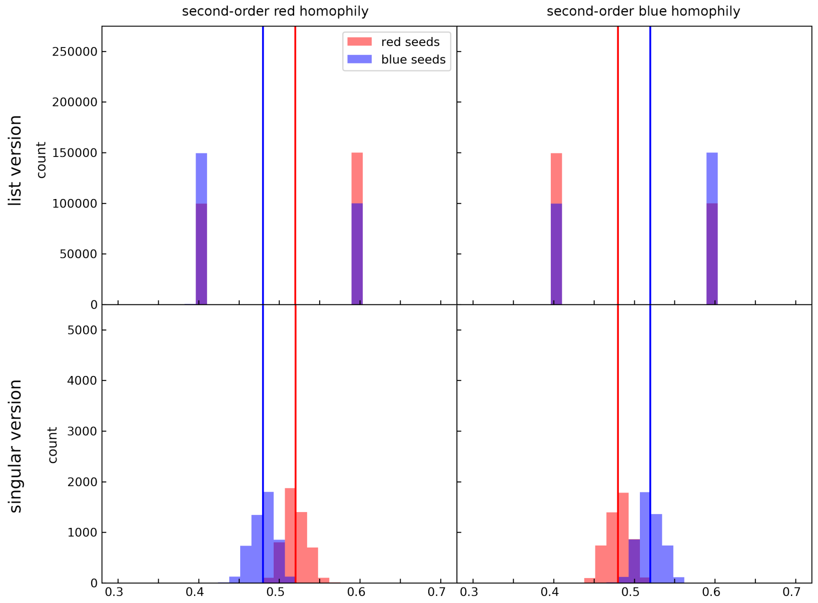

Figure S1: We see the gap in both the list and the singular version. Note that the means and the gaps are the same between versions, but that's not usually the case. The networks for each case were constructed separately.

5 Full derivation of list version gap size formula for a special case

We start with n nodes, k red nodes, $r = \frac{k}{r}$, sets R and B for red and blue nodes, degrees $d \in [0, n]$ for $i \in R$ and $c \in [0, n]$ for $j \in B$. First-order homophily h_i of $i \in R$ is distributed around mean λ_R with standard deviation σ_R, but then limited to $[0, 1]$ (clipping). Similarly, first-order homophily p_j of $j \in B$ is distributed around mean λ_B with standard deviation σ_B, but then limited to $[0, 1]$ (clipping). We observe that the expected number of edges between red and blue nodes is equal to $\sum_{i \in R} d_i(1 - h_i) = d \sum_{i \in R}(1 - h_i)$ and to $\sum_{j \in B} d_j(1 - h_j) = c \sum_{j \in B}(1 - h_j)$. This means that setting all parameters freely isn't possible. We can solve for c, for example:

$$c = d\frac{\sum_{i\in R}(1-h_i)}{\sum_{j\in B}(1-h_j)}$$

We can see how c would respond to changes in λ_R and σ_R given fixed values of everything else in Figure S2.

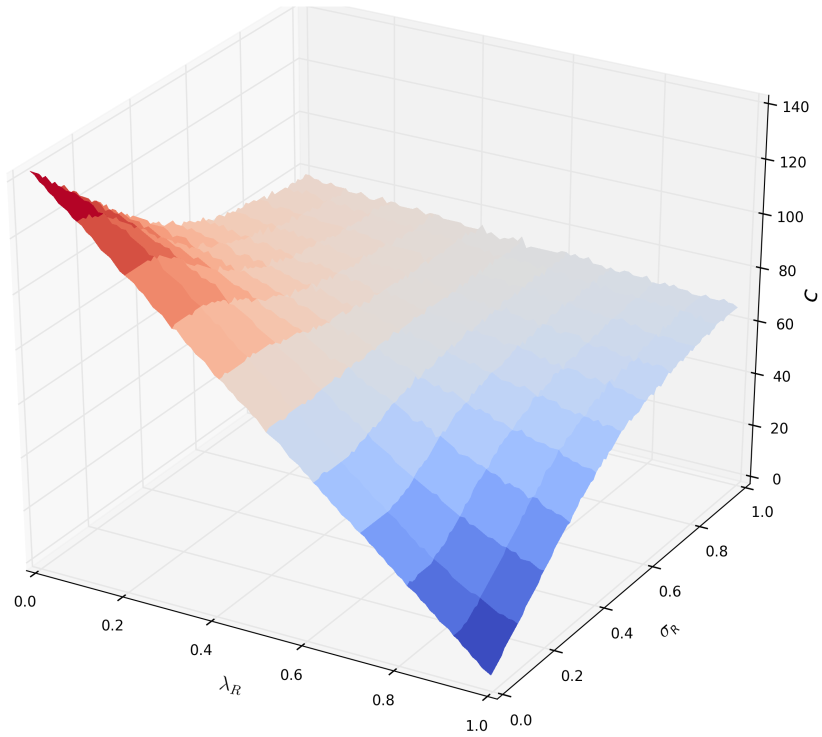

Figure S2: c as function of λ_R and σ_R. c changes due to the balance equation and clipping. The constant parameters are $n = 10000, d = 100, r = 0.5, \lambda_B = 0.3, \sigma_B = 0.15$.

Note that as we increase σ_R more clipping occurs and the first-order homophily of red nodes sample resembles a uniform distribution with sample mean = 0.5, regardless of the given λ_R.

A red person with homophily h_i would be counted $h_i d$ times in red nodes' second-order homophily values, and $(1 - h_i)d$ times in blue nodes' second-order homophily values. So, the total number of points in the red histogram would be $\sum_{i\in R} h_i d$ and in the blue histogram $\sum_{i\in R}(1 - h_i)d$.

So the mean of the red distribution would be:

$$\mu_R^{(R)} = \frac{\sum_{i \in R} h_i^2 d}{\sum_{i \in R} h_i d} = \frac{\sum_{i \in R} h_i^2}{\sum_{i \in R} h_i}$$

And the mean of the blue distribution would be:

$$\mu_B^{(R)} = \frac{\sum_{i \in R} h_i(1-h_i)d}{\sum_{i \in R}(1-h_i)d} = \frac{\sum_{i \in R} h_i(1-h_i)}{\sum_{i \in R}(1-h_i)}$$

If all h_i for $i \in R$ are equal to h, the equations become:

$$\mu_R^{(R)} = \frac{|R|h^2}{|R|h} = h$$

$$\mu_B^{(R)} = \frac{|R|h(1-h)}{|R|(1-h)} = h = \mu_R^{(R)}$$

Meaning there is no gap.

If h_i's are not identical ($\sigma_R \neq 0$), it is more difficult to deal with $\mu_R^{(R)}$ and $\mu_B^{(R)}$. We can normalize all the homophily values to have mean 0 and variance 1 by subtracting λ_R and dividing them by σ_R (the resulting homophily values would be denoted H_i). Then:

$$\sum_{i \in R} H_i^2 = \sum_{i \in R} \frac{(h_i - \lambda_R)^2}{\sigma_R^2} = \frac{1}{\sigma_R^2}\left(\sum_{i \in R} h_i^2 - 2\lambda_R \sum_{i \in R} h_i + \sum_{i \in R} {\lambda_R}^2\right) =$$

$$\frac{1}{\sigma_R^2}\left(\sum_{i \in R} h_i^2 - 2\lambda_R |R| \lambda_R + |R|{\lambda_R}^2\right) = \frac{1}{\sigma_R^2}\left(\sum_{i \in R} h_i^2 - |R|{\lambda_R}^2\right) =$$

$$\frac{\sum_{i \in R} h_i^2}{\sigma_R^2} - \frac{|R|{\lambda_R}^2}{\sigma_R^2} = \sum_{i \in R} H_i^2$$

Then the numerator of $\mu_R^{(R)} =$

$$\sum_{i \in R} h_i^2 = \sigma_R^2 \sum_{i \in R} H_i^2 + |R|{\lambda_R}^2$$

$\sum_{i \in R} H_i^2$ is a chi-square-distributed variable whose mean equals $|R|$. Then:

$$\sum_{i \in R} h_i^2 = |R|(\sigma_R^2 + {\lambda_R}^2)$$

Then

$$\mu_R^{(R)} = \frac{\sum\limits_{i \in R} h_i^2}{\sum\limits_{i \in R} h_i} = \frac{|R|(\sigma_R^2 + {\lambda_R}^2)}{|R|\lambda_R} = \frac{\sigma_R^2 + {\lambda_R}^2}{\lambda_R} = \lambda_R + \frac{\sigma_R^2}{\lambda_R}$$

$$\mu_B^{(R)} = \frac{\sum\limits_{i \in R} h_i(1 - h_i)}{\sum\limits_{i \in R}(1 - h_i)} = \frac{\sum\limits_{i \in R} h_i - \sum\limits_{i \in R} h_i^2}{|R| - \sum\limits_{i \in R} h_i} =$$

$$\frac{|R|\lambda_R - |R|(\sigma_R^2 + {\lambda_R}^2)}{|R| - |R|\lambda_R} = \frac{\lambda_R - (\sigma_R^2 + {\lambda_R}^2)}{1 - \lambda_R} = \lambda_R - \frac{\sigma_R^2}{1 - \lambda_R}$$

This also seems consistent with the data. Now we'd like to prove that $\mu_R^{(R)} > \mu_B^{(R)}$. This is clear, because $\mu_R^{(R)} > \lambda_R$ and $\mu_B^{(R)} < \lambda_R$ (if $\sigma_R > 0$).

The gap would be equal to:

$$g = \frac{\sigma_R^2}{\lambda_R} + \frac{\sigma_R^2}{1 - \lambda_R}$$

In general, because of the denominators and the square, g would be > 0 for $0 < \lambda_R < 1$ and $\sigma_R > 0$ (and also $\sigma_R < 0$, but that's not important). g would be defined for $0 < \lambda_R < 1$.

Note: this might be an abuse of notation because we initially derived this for $\sigma_R \neq 0$, but if $\sigma_R = 0$, $g = 0$.

6 Examples where the Friendship and the Homophily Paradoxes co-occur and don't

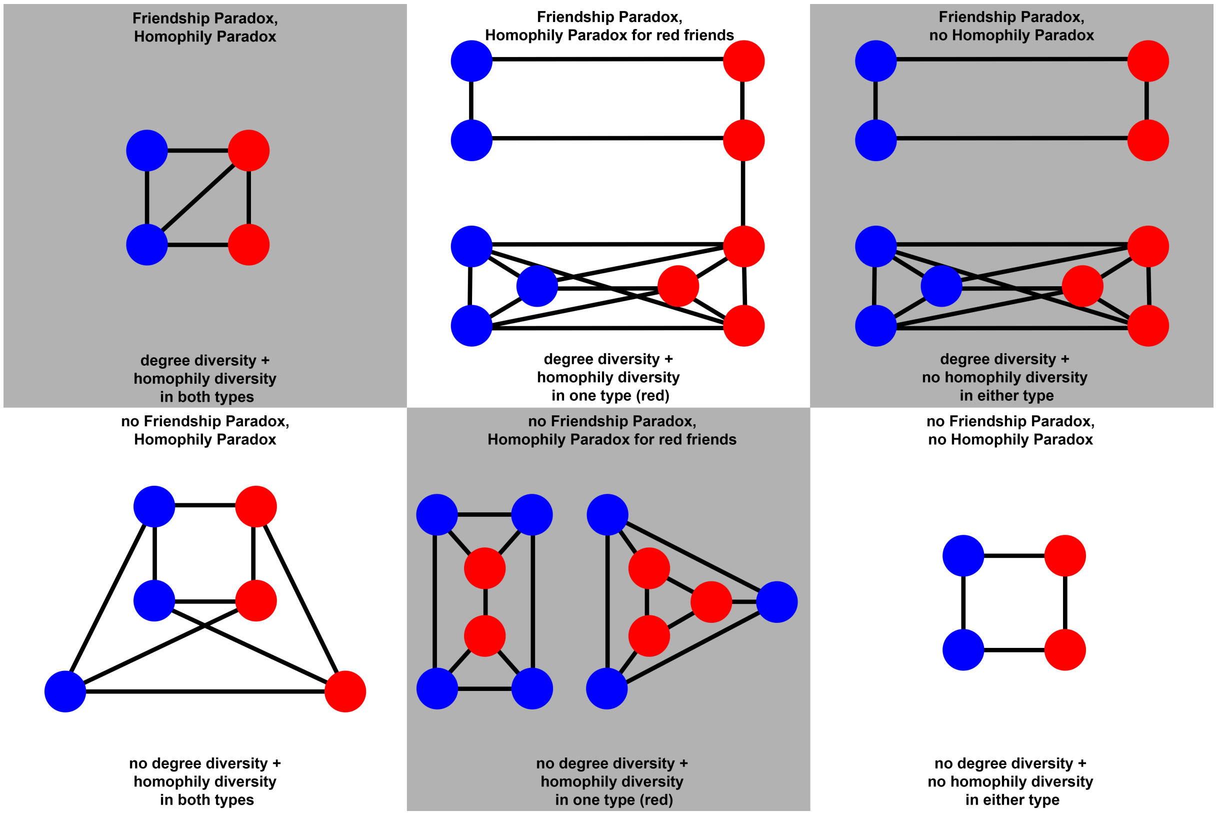

Figure S3: Example networks where the Friendship and the Homophily Paradoxes co-occur and don't.

References

1. Kramer, J. B., Cutler, J. & Radcliffe, A. The multistep friendship paradox. *The American Mathematical Monthly* **123**, 900–908 (2016).
